# Hypoxia and TGF-β1 induced PLOD2 expression improve the migration and invasion of cervical cancer cells by promoting epithelial-to-mesenchymal transition (EMT) and focal adhesion formation

**DOI:** 10.1186/s12935-017-0420-z

**Published:** 2017-05-12

**Authors:** Feifei Xu, Jialu Zhang, Guolin Hu, Lei Liu, Weijiang Liang

**Affiliations:** 1 Department of Oncology, Nanfang Hospital, Southern Medical University, Guangzhou, 510515 China; 2 Department of Oncology, The Sixth Affiliated Hospital of Guangzhou Medical University, Qingyuan People’s Hospital, Qingyuan, 511518 China

**Keywords:** PLOD2, Cervical cancer cells, Migration, Invasion, EMT, Focal adhesion

## Abstract

**Background:**

Intra-tumoral hypoxia and increases in extracellular level of transforming growth factor β1 (TGF-β1), which are common findings in cancer, are associated with an increased risk of metastasis and mortality. Moreover, metastasis is the leading cause of death of patients with cervical cancer. PLOD2 is an intracellular enzyme required for the biogenesis of collagen and its expression can be induced by hypoxia and TGF-β1. Specifically, PLOD2 is up-regulated in several types of cancer, including cervical cancer, and is associated with cancer metastasis. Thus, in this research, we aimed to investigate the role of PLOD2 in the motility of cervical cancer cells and to show the molecular mechanism underlying this effect.

**Methods:**

siRNA was used to knockdown PLOD2 in the cervical cancer cell lines HeLa and SiHa. The ability of cells to migrate and invade, their adhesion to type I collagen, and their capacity for epithelial-to-mesenchymal transition (ΕΜΤ) and focal adhesion formation were analyzed. Gene expression changes were validated by qRT-PCR, Western blotting and Immunocytochemistry. The morphological status of cells was examined using phalloidin staining. Differences in PLOD2 expression among patients with cervical cancer were identified by referring to public databases, including Oncomine and TCGA.

**Results:**

Hypoxia and TGF-β1 enhanced the expression of PLOD2 in HeLa and SiHa cells, and knockdown of PLOD2 inhibited cell motility and EMT. Moreover, the depletion of PLOD2 attenuated hypoxia-mediated cell migration and invasion and inhibited TGF-β1-induced phenotypic EMT-like changes by preventing β-catenin from entering the nucleus. In addition, PLOD2 depletion decreased cell adhesion to extracellular collagen by inhibiting the formation of focal adhesions. Moreover, a database analysis showed that PLOD2 expression is associated with human cervical cancer progression.

**Conclusions:**

Overall, our results indicated that hypoxia- and TGF-β1-induced PLOD2 expression promotes the migratory, invasive and adhesive capacities of cervical cancer cells by participating in TGF-β1 induced EMT and the formation of focal adhesions.

**Electronic supplementary material:**

The online version of this article (doi:10.1186/s12935-017-0420-z) contains supplementary material, which is available to authorized users.

## Background

Cervical cancer is the third most common cancer in women worldwide [1], and the incidence rate and mortality of this disease are higher in developing countries than in developed countries [2]. Although cisplatin-based concurrent chemo-radiotherapy improves the overall survival, progression-free survival, and recurrence rate in patients with locally advanced disease [3], approximately 50% of patients with locally advanced disease are expected to relapse within the first 2 years after initial treatment [4]. Specifically, metastases to the pelvic lymph nodes and especially the para-aortic lymph nodes are associated with poorer survival [5]. Relapse within a previously irradiated field and primary metastatic disease are considered incurable [4, 6]. Therefore, preventing lymph node and distant metastases remains an important yet unresolved therapeutic goal for these patients. However, biomarkers to accurately predict metastases are currently not available.

Hypoxia is an important characteristic feature of solid tumours, such as cervical cancer, and it has been associated with the presence of lymph node metastases and higher rates of distant dissemination [7–9]. As shown in [10], hypoxia indicates a poor prognosis in irradiated cervical carcinomas, which suggests that hypoxia is an important marker of progression in cervical cancer. Therefore, we examined the utility of hypoxia-induced genes as biomarkers for predicting metastasis in cervical cancer. The long-established hypoxia-induced gene [11–13] PLOD2 is histologically over-expressed in sarcoma, glioblastoma, breast cancer and hepatocellular carcinoma [14–17], and it is an independent prognostic factor in glioblastoma and hepatocellular carcinoma [15, 17]. However, little is known about the role of this gene in cervical cancer.

PLOD2 primarily initiates the lysine hydroxylation of collagen molecules [18–20]. The resultant hydroxylysyl groups are attachment sites for carbohydrates in collagen and are consequently critical for the stability of intermolecular crosslinks [21]. Notably, PLOD2 is located between 3q21 and 3q26, a chromosomal region previously reported to be amplified in cervical cancer [22]. An analysis of gene expression profiles using oligonucleotide microarrays first identified PLOD2 as an aberrantly up-regulated gene [23]. Comparisons between different types of samples among studies subsequently suggested that PLOD2 is up-regulated in squamous cell carcinomas relative to cells of the normal cervix and high-grade squamous intra-epithelial lesion [24] and could be associated with the invasiveness of cervical carcinoma cells [25].

This report is designed to investigate the effects of PLOD2 on cell migration or invasion and further evaluate its role in the hypoxia-induced increase in cell motility and the TGF-β1-mediated EMT of cervical cancer cells.

## Methods

### Cell lines and culture

HeLa (cervical adenocarcinoma) and SiHa (squamous carcinoma of the cervix) cell lines were obtained from the Department of Oncology at Southern Medical University (Guangzhou, China). Both cell lines were maintained in Dulbecco’s Modified Eagle’s Medium (DMEM, Gibco, USA) supplemented with 10% foetal bovine serum (FBS) (Biological Industries, Israel), 100 U/ml penicillin, and 100 U/ml streptomycin (Gibco, USA) in a humidified incubator containing 5% CO_2_ at 37 °C.

### RNA interference

HeLa and SiHa cells at approximately 30% confluence were transfected with 10 nM PLOD2 siRNA or scramble siRNA purchased from Invitrogen (Life Technologies, USA) using Lipofectamine 3000 Reagent (Life Technologies, USA) according to the manufacturer’s protocol. PLOD2 knockdown was validated by quantitative Real-Time PCR and by Western blotting 24 and 48 h after transfection, respectively.

### Treatment of minoxidil, cobalt chloride and recombinant human TGF-β1

Cells (2 × 10^5^) were seeded in six-well plates and cultured until they reached 30% confluence. For the treatment of minoxidil, the medium was then replaced with DMEM (high-glucose) supplemented with 10% FBS and 0.5 mM minoxidil. For the treatment of cobalt chloride, the medium was then replaced with 150 or 200 μM cobalt chloride to investigate the optimum concentration. Meantime, cells with the same medium with an equal volume of PBS were set as blank control. The cells of all groups were then cultured for 24, 48 and 72 h to investigate the effect of minoxidil or cobalt chloride on PLOD2 expression over time. The medium containing minoxidil or cobalt chloride was replaced daily. For all experiments, minoxidil and cobalt chloride were directly dissolved in PBS and filtered through a 0.22 μm filter (Merck Millipore, USA) before use.

To examine the effect of TGF-β1, 2 × 10^5^ control cells (mock or si-scramble) or PLOD2-siRNA cells were seeded into each well of a six-well plate and cultured in DMEM (high-glucose) supplemented with 10% FBS overnight. The cells were then treated with recombinant human TGF-β1 (Peprotech, USA) in FBS-free medium at concentrations ranging from 0.2 to 10 ng/ml to select the optimal concentration. The medium was replaced every other day. After treatment for 72 h, the cells were collected for total protein extraction.

### Real-time reverse transcription quantitative PCR

Total RNA was isolated from cells using TRIzol reagent (TAKARA Biotechnology, Japan), and total mRNA (1 μg) was reverse transcribed to cDNA using PrimeScript RT Master Mix (TAKARA Biotechnology, Japan). Transcript expression was assessed by subjecting synthesized cDNA to quantitative PCR using an Applied Biosystems 7500 Real-Time PCR System (Thermo Fisher Scientific, USA). Target cDNA amplification was measured using SYBR Premix Ex Taq II (TAKARA Biotechnology, Japan) for human PLOD2, GAPDH, CDH1, CTNNB1, and VIM. The fold-change in the expression of each target mRNA relative to GAPDH was calculated using the CT (2^−ΔΔCT^) method. The experiments were performed at least three times, and the resultant data were statistically analysed. The following primers were used for qRT-PCR: PLOD2 forward, CATGGACACAGGATAATGGCTG and reverse, AGGGGTTGGTTGCTCAATAAAAA; CDH1 forward, CCCGGGACAACGTTTATTAC and reverse, GCTGGCTCAAGTCAAAGTCC; CTNNB1 forward, GAATATCTGTAATGGTAC and reverse, CTATAACTTAACACTACG; VIM forward, CTCCTACAAGATTTAGAA and reverse, GATTTATTGAAGCAGAAC; and GAPDH forward, GAAGGTGAAGGTCGGAGTC and reverse, GAAGATGGTGATGGGATTTC.

### Western blotting assays

Whole-cell lysates were prepared in RIPA lysis buffer containing 1 mM phenylmethylsulfonyl fluoride (PMSF) and protease inhibitor cocktail (Cell Signaling Technology, USA). The concentration of protein was quantified with the Bradford Method using Coomassie Blue Staining Solution (Beyotime, China). Total protein was then separated by 8% SDS-PAGE, transferred to polyvinylidene difluoride (PVDF) membranes and probed with primary antibodies against HIF-1α (Abclonal, USA), PLOD2, β-catenin (Proteintech, USA), E-cadherin (Genetex, USA), FAK, p-FAK (Abcam, USA), AKT, phosphorylated-AKT, MMP2, MMP9 (Abcam, USA) and Vimentin (Cell Signaling Technology, USA). The bands were detected using Enhanced Chemiluminescence (ECL) (Bio-rad, USA) or the Odyssey IR Imaging System (LI-COR Bioscience, USA).

### Immunocytochemistry and phalloidin staining

Cells cultured on 15 mm coverslips were washed twice in phosphate-buffered saline (PBS) and fixed with 4% paraformaldehyde at 4 °C for 30 min. After fixation, the cells were permeabilized using 0.1% Triton X-100 for 10 min and blocked with 5% bovine serum albumin for 30 min at room temperature. The samples were then incubated with primary antibodies at 4 °C overnight, followed by incubation with Alexa Fluor 594-conjugated goat anti-rabbit secondary antibody for 1 h at room temperature. To analyse p-FAK expression, Alexa Fluor 488 phalloidin was used at the same time with secondary antibody (Thermo Fisher Scientific, USA). The coverslips were then fixed on slides using Fluoroshield Mounting Medium with DAPI (Abcam, USA), which contains DAPI for the counterstaining of nucleus. Fluorescent photos were captured by a fluorescence microscope (Olympus BX-51, Japan) with oil immersion lens at 1000× magnification.

### Migration and invasion assays

Migration assays were conducted using 24-well Boyden chambers containing inserts (8 μm pores; BD Biosciences, USA). Invasion assays were performed similarly using matrigel-coated inserts (BD Bioscience, USA). The lower chamber was filled with medium containing 10% serum, whereas the top chamber contained 1 × 10^5^ HeLa cells or 5 × 10^4^ SiHa cells suspended in medium without serum. The plates were incubated at 37 °C in 5% CO_2_ for 24 h. After migration, non-migrated cells remaining on the top of the inserts were removed with cotton swabs, whereas the cells that had migrated to the underside of the membrane was fixed with paraformaldehyde and stained with 1% crystal violet. Photos were captured with a fluorescence microscope (Olympus IX71, Japan). Migrated cells on each insert were counted in six randomly selected high-power fields (under a 20× objective lens) and quantified using the ImageJ software.

Scratch assays were conducted on confluent cells and seeded in six-well plates. After the cells had adhered to the plates, the wells were gently scratched with straight lines using 20 μl sticks. When cells migrated into the wounds, the area decreased, during which cells were imaged every 12 for 48 h under a microscope (OLYMPUS IX71, Japan). For each experimental condition, the areas devoid of cells in six unique fields (under a 20× objective lens) were measured using ImageJ software.

### Adhesion assays

Cells (2.5 × 10^5^) were plated in serum-free medium on matrigel (BD Biosciences, USA) and type I collagen (Corning, USA)-coated 96-well plates and allowed to adhere. 2 h later, non-adherent cells were removed by washing the plates twice with PBS. Adherent cells were photographed with a microscope (OLYMPUS IX71, Japan) under a 20× objective lens, and cells were counted using the ImageJ software.

### Statistical analysis of PLOD2 expression in cervical cancer

The gene expression levels of a cervical cancer data set (Zhai Cervix) retrieved from the Oncomine database (http://www.oncomine.org) were analysed. A cervical cancer dataset (TCGA, Provisional) that contained 307 total patients was queried. Specifically, genetic and transcriptional changes in PLOD2 expression were assessed, and these changes were correlated with overall survival using a Kaplan–Meier analysis by cBioPortal (http://www.cbioportal.org), a tool developed by the Computation Biology Center at Sloan Kettering.

### Statistical analysis

Data are represented as the mean ± SD. Unpaired two-tailed Student’s t tests were conducted to evaluate the differences between control and experimental groups. Significance is indicated by the presence of an asterisk (*p* < 0.05 is indicated by “**”, *p* < 0.01 is indicated by “***”, and *p* < 0.1 is indicated by “*”). The quantified data shown represent at least three independent experiments. SPSS Statistics was used to conduct all statistical analyses, and the Graphpad Prism software was used to generate statistical charts.

## Results

### Depletion of PLOD2 inhibits mobility of cervical cancer cells

**Fig. 1:**
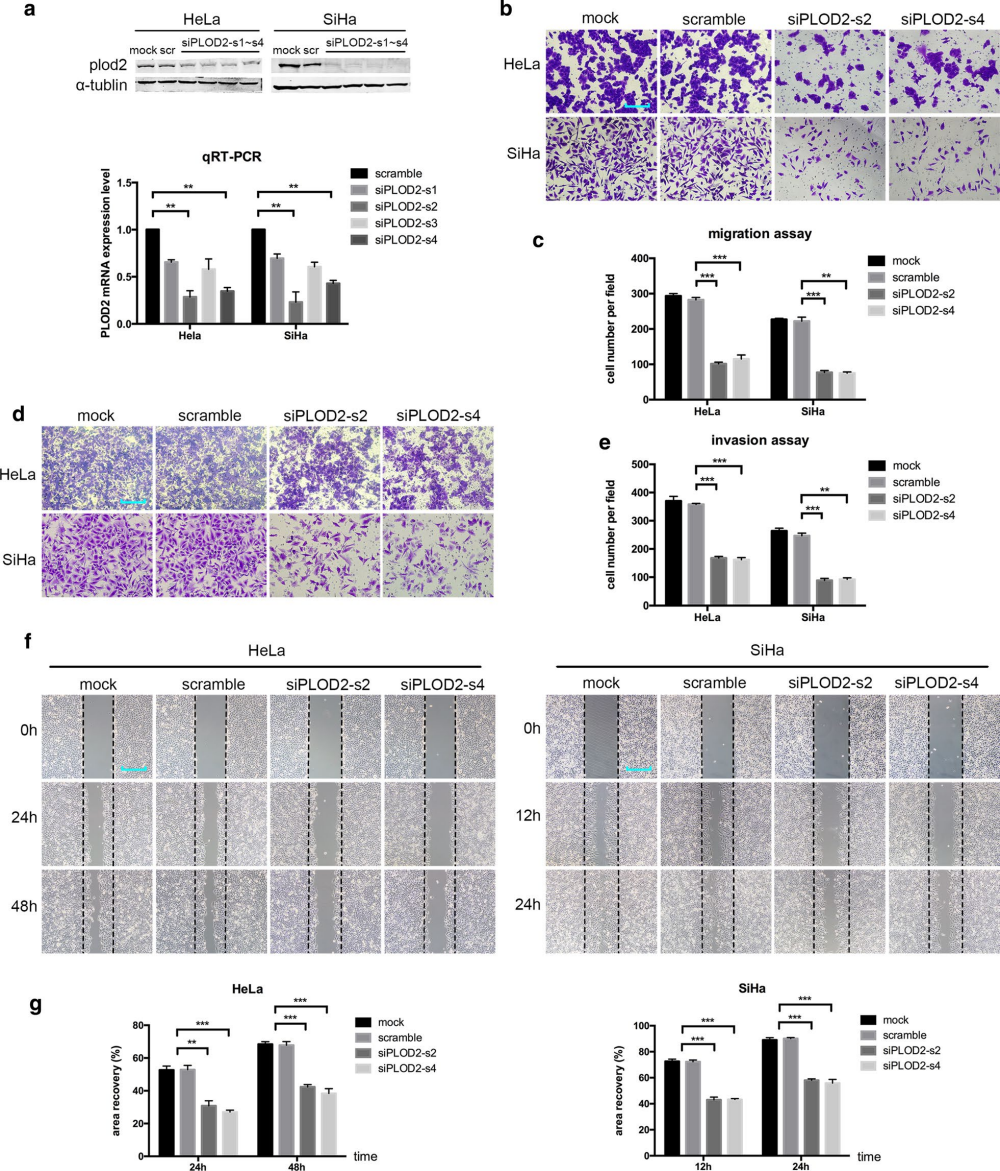
Knockdown of PLOD2 inhibits cell migration and invasion. **a** Knockdown of PLOD2 in HeLa and SiHa cells. Cells were transfected with si-scramble or siPLOD2 and then subjected to a Western blot analysis and qRT-PCR; α-tubulin and GAPDH were used as loading controls, respectively. **b**, **d** Depletion of PLOD2 inhibited cell migration and invasion. Si-scramble and siPLOD2 cells were subjected to transwell migration (**b**) and matrigel-coated invasion assays (**d**). Photos were captured under an ×200 objective lens. **c**, **e** Statistical analysis of migration (**c**) and invasion (**e**) based on the mean ± SD of at least three independent experiments. *p* values were obtained using Student’s t test. *p* < 0.05 is indicated by “**”, and *p* < 0.001 is indicated by “***”, si-scramble versus siPLOD2-s2 or siPLOD2-s4. **f** Scratch assays corroborated the decrease in HeLa and SiHa cell migration after the knockdown of PLOD2. Images were captured at ×200 magnification. **g** Statistical data were obtained as described above

As previously reported, PLOD2 expression strongly correlates with metastasis in several types of cancer, such as sarcoma, breast cancer and lung cancer [14, 16, 26]. Thus, we hypothesized that PLOD2 controls the migration and invasion of cervical cancer cells. To test this hypothesis, HeLa and SiHa cells were transfected with siRNA targeting PLOD2 and non-targeting (scramble) siRNA, and the former effectively suppressed the protein and mRNA expression of PLOD2, as measured using Western blotting and quantitative real-time PCR (Fig. 1a). Among the four PLOD2 siRNAs, siPLOD2-s2 and siPLOD2-s4 exhibited the maximum inhibition efficiency. Thus, they were selected for subsequent studies and are henceforth referred to as siPLOD2-s2 and siPLOD2-s4 in figures. To assess the biological significance of PLOD2 on cell mobility, we conducted transwell migration assays and matrigel-coated invasion assays, which showed that the siRNA-mediated knockdown of PLOD2 significantly decreased the migratory and invasive capacities of both HeLa and SiHa cells (Fig. 1b, d). A statistical analysis indicated that these capacities significantly differed between the scramble group and siPLOD2 group (Fig. 1c, e). Moreover, scratch assays were conducted to investigate the effects of PLOD2 on the migratory behaviours of cells in vitro. The degree of wound healing was assessed every 12 h using a microscope, and representative pictures obtained with a 200× objective lens at 24 and 48 h for HeLa cell and 12 and 24 h for SiHa cells are shown (Fig. 1f). The statistical analysis showed results consistent with those presented above (Fig. 1g).

### Minoxidil-mediated suppression of PLOD2 impairs the migration and invasion of cells

**Fig. 2:**
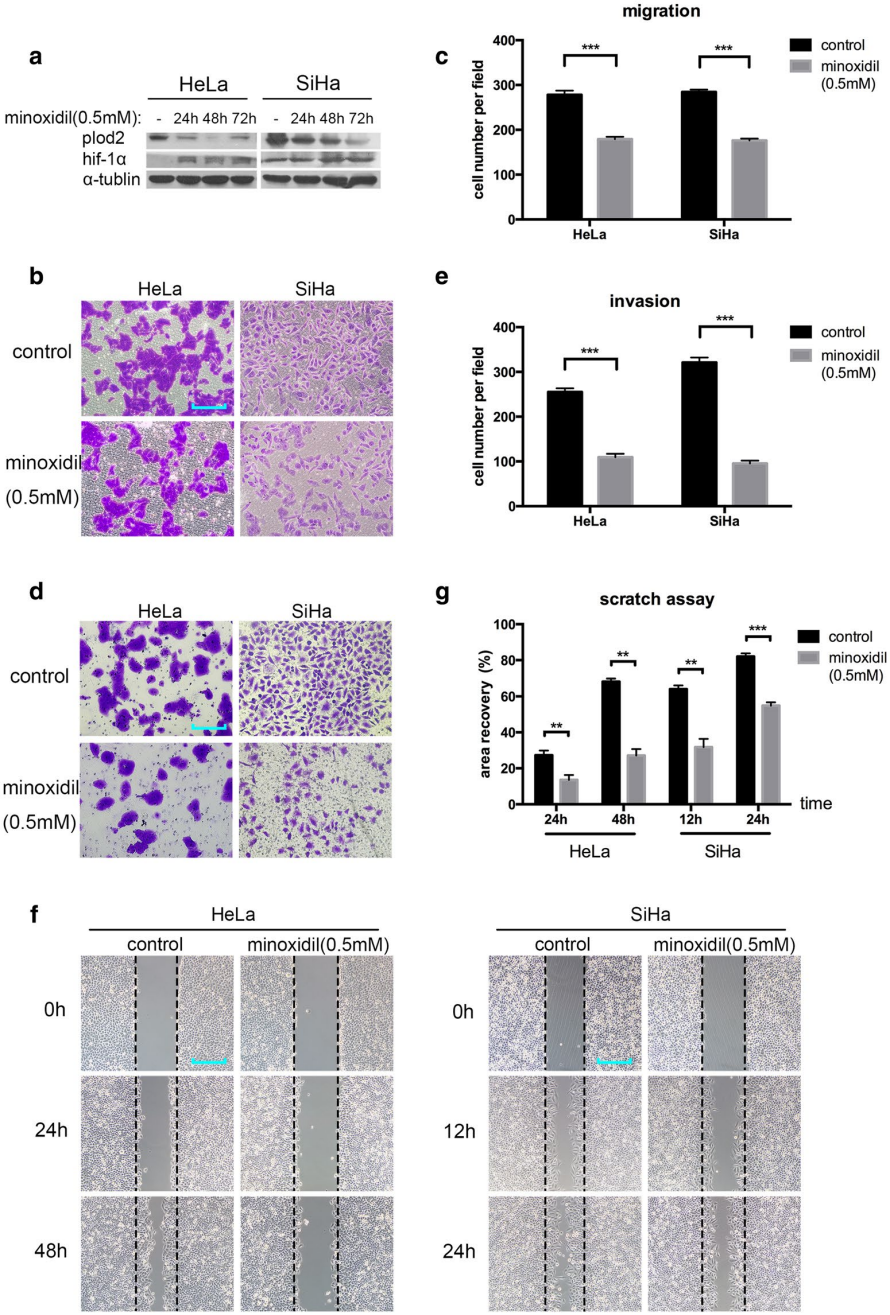
Minoxidil-mediated inhibition of PLOD2 attenuates cell migration and invasion. **a** Inhibition of PLOD2 by minoxidil (0.5 mM) in HeLa and SiHa cells. Cells were treated with minoxidil (0.5 mM) for 24, 48 or 72 h and then subjected to a Western blot analysis; α-tubulin was used as a loading control. **b**, **d** The inhibition of PLOD2 invasion and migration. Cells treated with minoxidil (0.5 mM) for 48 h and control cells were subjected to transwell migration (**b**) and matrigel-coated invasion assays (**d**). Photos were captured under a ×200 objective lens. **c**, **e** Statistical analysis of migration (**c**) and invasion (**e**) based on the mean ± SD of at least three independent experiments. *p* values were obtained using Student’s t test. *p* < 0.05 is indicated by “**”, and *p* < 0.001 is indicated by “***”. **f** Scratch assays further supported that minoxidil (0.5 mM) treatment inhibited the migration of HeLa and SiHa cells. Photos were captured under a ×200 objective lens. **g** Statistical data were obtained as described above

To corroborate that PLOD2 is required for cervical cancer cells to disseminate, we used a previously described pharmacologic inhibitor of PLOD2 expression, minoxidil [27]. Specifically, minoxidil treatment (0.5 mM) significantly reduced HT-1080 sarcoma cell migration and notably decreased the number of pulmonary metastases formed subcutaneously injected sarcoma cells in nude mice [14]. In our study, minoxidil treatment (0.5 mM) for 48 h significantly reduced HeLa and Siha cell migration (Fig. 2b) and invasion (Fig. 2d) and significantly decreased the PLOD2 protein levels, as shown by Western blotting (Fig. 2a). A statistical analysis validated that this decrease was significant when comparing the control group and minoxidil-treated group (Fig. 2c, e).

Moreover, scratch assays showed that minoxidil treatment inhibited wound healing compared with control cells (Fig. 2f). Statistical analysis presented the consistent results with above (Fig. 2g). These data demonstrate the utility of minoxidil as a treatment for pre-metastatic cervical cancer.

### PLOD2 mediates the HIF-1α-dependent promotion of cell migration and invasion

Metastasis is a complex multistep process wherein tumour cells are driven, in part by a lack of oxygen and nutrients, to abandon their tissue of origin and colonize distant sites [28]. In cervical cancer, adjuvant chemo-radiotherapy is necessary for patients who have developed postoperative reoccurrence, during which the tumour may experience low-oxygen conditions. HIF-1α is a major regulator in helping cells survive hypoxia and has been confirmed to promote metastasis by regulating tumour cell migration and invasion.

Hypoxia has been shown to induce the expression of PLOD2 in various cell types [11–14, 16, 29], including cervical cancer cells [11]. Specifically, the HIF-1α-dependent induction of lysyl hydroxylase activity is required for breast cancer and sarcoma cell migration [14, 16]. In our study, minoxidil increased the HIF-1α levels, but not cell migration (Fig. 2a), suggesting that the HIF-1α-dependent induction of PLOD2 is required for cell motility.

**Fig. 3:**
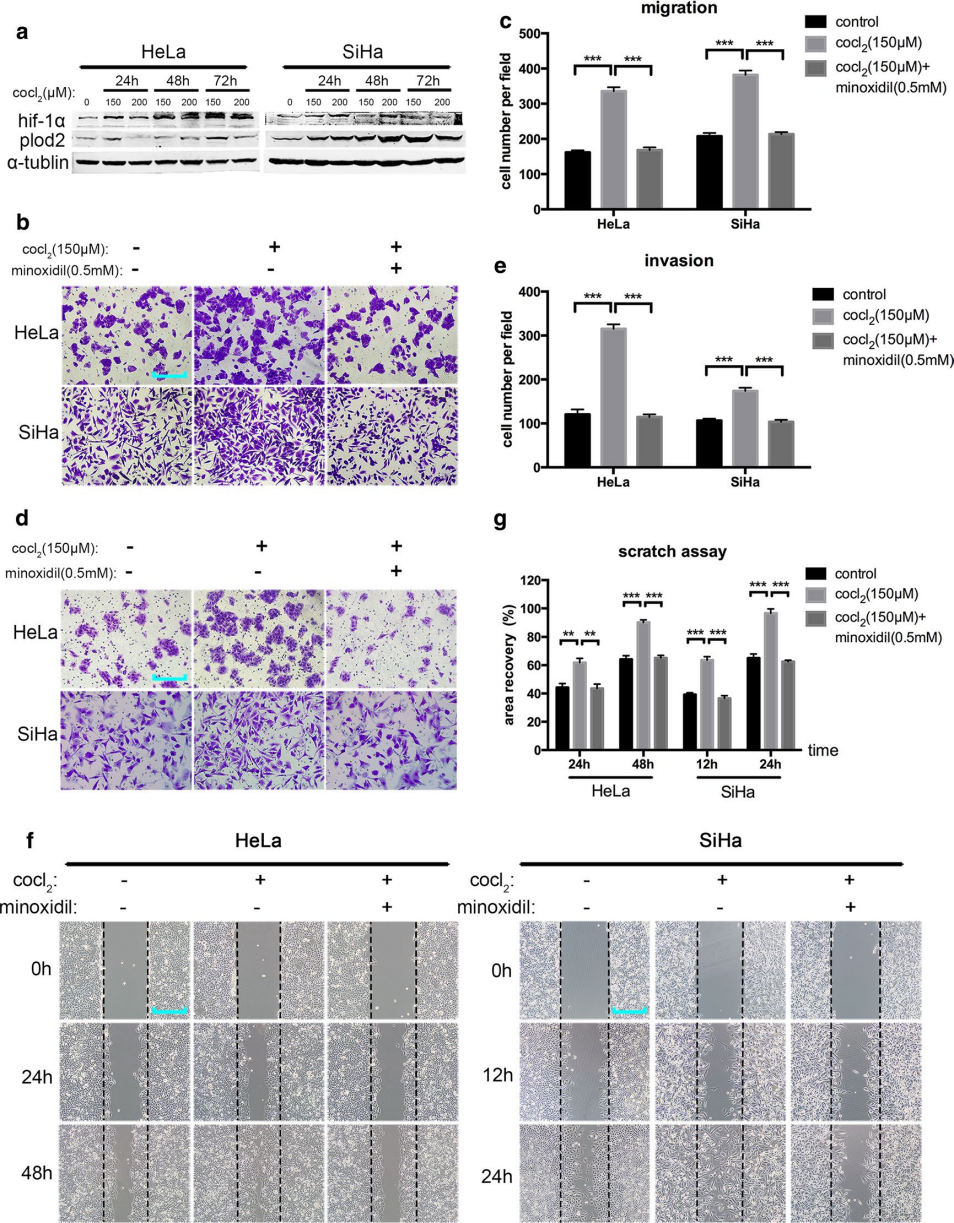
PLOD2 mediates the HIF-1α-stimulated migration and invasion of cells. **a** The effect of hypoxia was mimicked by cobalt chloride treatment (150 and 200 μM) for 24–72 h. Western blot showing an increase in PLOD2 and concurrent HIF-1α induction, with a maximum effect on 72 h. **b**, **d** Hypoxic cells pre-treated with cobalt chloride (150 μM) were then treated with minoxidil (0.5 mM) to inhibit PLOD2 expression. A migration assay (**b**) and matrigel-coated invasion assay (**d**) were conducted to compare the migratory and invasive ability of control cells, hypoxic cells, and hypoxic and PLOD2-inhibited cells. Photos were captured under a ×200 objective lens. **c**, **e** Statistical analyses of migration (**c**) and invasion (**d**) based on the mean ± SD of triplicate independent experiments. *p* values were obtained using Student’s t test. *p* < 0.05 is indicated by “**”, and *p* < 0.001 is indicated by “***”. **f** Scratch assays were conducted to support the results shown in **b**. Photos were captured under a ×200 objective lens. **g** Statistical analyses of scratch assay data (**f**) were conducted as described above

To confirm this dependence, we pre-treated HeLa and SiHa cells with cobalt chloride (150 and 200 μΜ) for 24–72 h to stabilize the structure of HIF-1α and simulate hypoxia. Western blotting results showed that PLOD2 expression increased, and HIF-1α expression was induced (Fig. 3a). Hypoxic cells were then treated with 0.5 mM minoxidil to inhibit the expression of PLOD2, and transwell and matrigel-coated transwell assays showed that this suppression of PLOD2 attenuated HIF-1α mediated increases in cell motility to a level identical to that of control cells (Fig. 3b, d). A statistical analysis validated that the difference between the hypoxia group and hypoxia and PLOD2-inhibited group was significant (Fig. 3c, e). Moreover, a scratch assay was conducted to investigate the effects of PLOD2 on the migratory behaviours of hypoxia cells in vitro (Fig. 3f). A statistical analysis produced results consistent with those presented above (Fig. 3g). These results demonstrate that the hypoxia-mediated malignant behaviour is partly due to increases in PLOD2 expression.

Tumour invasion and metastasis through biological barriers require the proteolytic degradation of extracellular matrix (ECM) components, which is principally mediated by the matrix metalloproteinase (MMP) family [30, 31]. Specifically, MMP-2 and MMP-9 are the most-studied MMPs in the context of cancer, including metastasis [32, 33]. Moreover, hypoxia stimulates the expression and activity of MMP-2 and MMP-9 via an HIF-1α dependent process [34–36]. As shown in Fig. 3, inhibiting PLOD2 with minoxidil under hypoxic conditions impaired migration and invasion compared with control cells. Therefore, we focused on MMP-2 and MMP-9 when investigating the molecular mechanism by which PLOD2 reduces the migration and invasion of cervical cancer cells in hypoxic conditions. Specifically, we investigated the direct effect of PLOD2 on the expression of MMP-2 and MMP-9. However, knockdown of PLOD2 with siRNA did not reduce the protein expression levels of MMP-2 and MMP-9 (Additional file 1: Fig. S1a). Moreover, we explored the effect of PLOD2 on the HIF-1α-dependent induction of MMP-2 and MMP-9, but PLOD2 also failed to inhibit these proteins under hypoxic conditions (Additional file 1: Fig. S1b). Therefore, we proposed that PLOD2 impairs cell motility under hypoxic conditions not by interfering with the protein expression of MMP-2 and MMP-9 but by other molecular mechanisms, such as by activating MMP-2 and MMP-9 or by participating in the regulation of other metastasis-related genes.

### Suppression of PLOD2 promotes an epithelial phenotype in cervical cancer cells

HIF-1α is known to influence cell migration and invasion by modulating multiple intrinsic cell effectors, including the expression of Snail and Twist1, which are specific transcriptional regulators of EMT [37–39]. Moreover, transforming growth factor-β (TGF-β) induced EMT is well-established as an essential mechanism of cervical cancer progression [40]. During the metastatic progression of carcinoma, polarized epithelial tumour cells gain migratory and invasive characteristics in order to disseminate from primary tumour sites.

**Fig. 4:**
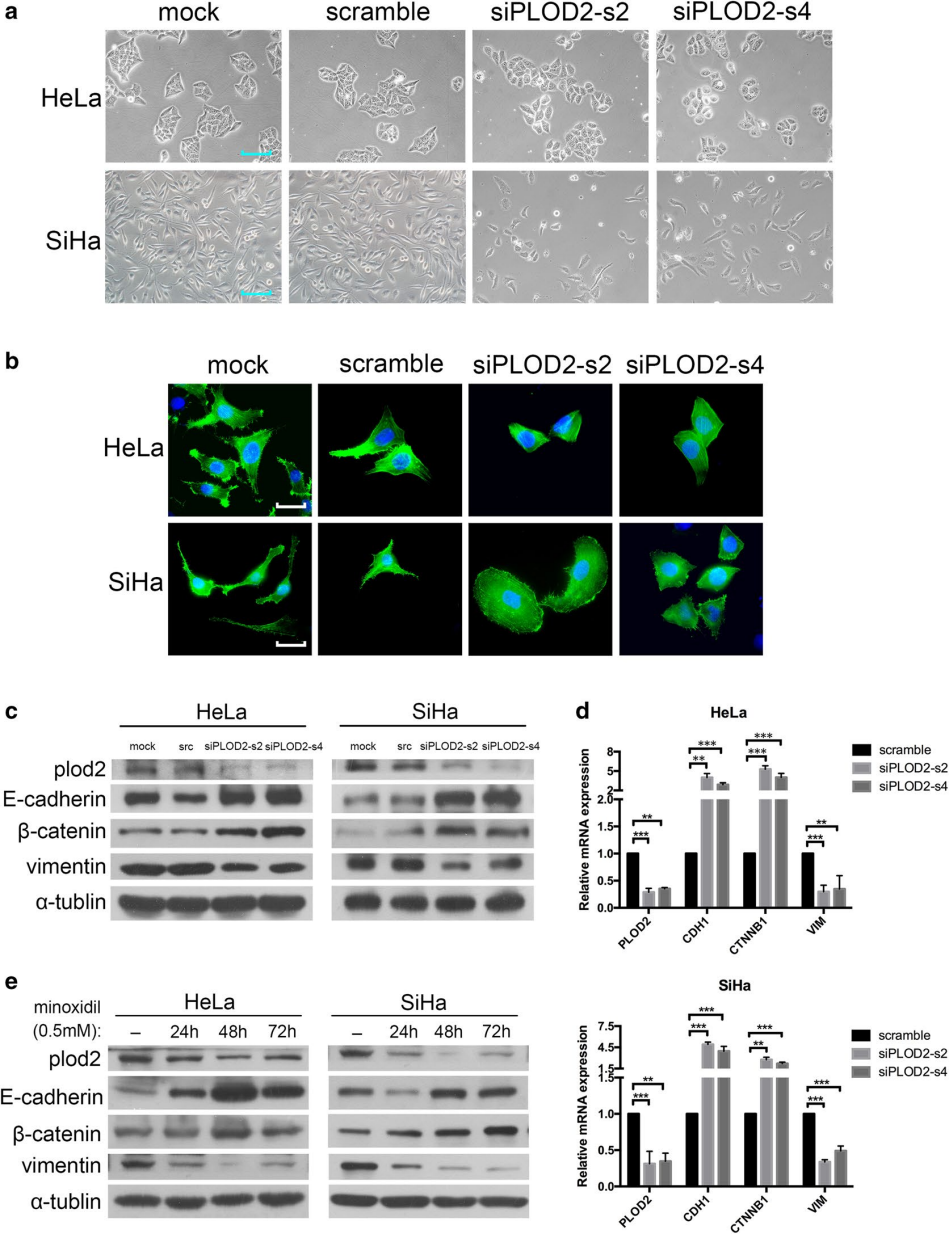
Knockdown of PLOD2 inhibits the morphologic and phenotypic EMT-like changes in cervical cancer cells. **a** PLOD2-silenced HeLa and SiHa cells exhibited a rounded shape. Representative phase-contrast images of control and siPLOD2 cells obtained at ×200 magnification are shown. **b** Phalloidin staining revealed minimal pseudopods in siPLOD2 cells, whereas control cells exhibited abundant and elongated pseudopods. Morphologic changes shown by phalloidin staining are consistent with **a**. Representative fluorescence images of control and siPLOD2 cells are shown. Photos were captured under an oil lens (×1000). **c**, **d** Knockdown of PLOD2 resulted in a gain of E-cadherin and β-catenin and a loss of vimentin. The protein and mRNA expression levels of E-cadherin, β-catenin, and vimentin were measured by Western blotting (**c**) and qRT-PCR (**d**); α-tubulin and GAPDH were used as loading controls, respectively. “src” in **c** indicates the scramble group. Data shown in **d** are based on the mean ± SD of triplicate independent experiments. *p* values were obtained using Student’s t test. *p* < 0.05 is indicated by “**”, and *p* < 0.001 is indicated by “***”. **e** Inhibition of PLOD2 by minoxidil (0.5 mM) led to a similar change in EMT molecular markers, as demonstrated by Western blotting

In our study, the knockdown of PLOD2 markedly affected the morphology of SiHa cells: the spindle- and fibroblast-like morphology switched to a cobblestone-like appearance, which is characteristic of epithelial cells. Correspondingly, the shape of HeLa cells changed from irregular polygons to round cells. Moreover, HeLa cells originally grew as spheroid-like cell clusters, whereas PLOD2-silenced cells tended to be mutually isolated (Fig. 4a). The morphological transition after PLOD2 depletion suggested that PLOD2 participates in the regulation of EMT.

The ability of cells to reconstruct and contact three-dimensional type I collagen gels is regarded as an in vitro measure of cell adhesion and invasion [41]. We stained cells with phalloidin to observe their morphology more clearly; control cells exhibited a branching morphology typical of invasive human cancer cells grown on collagen type I gels, whereas PLOD2 siRNA cells grew in a considerably different manner, showing little branching and remaining round (Fig. 4b).

In addition, an increase in the expression of mesenchymal molecular markers and a simultaneous decrease in the expression of epithelial markers are characteristic of EMT. In our work, Western blotting showed that the knockdown of PLOD2 contributed to a gain of epithelial markers, such as E-cadherin and β-catenin, and a loss of the mesenchymal marker vimentin (Fig. 4c). Quantitative RT-PCR showed similar changes in the mRNA levels of CDH1, CTNNB1, and VIM, which encode E-cadherin, β-catenin, and vimentin (Fig. 4d). These data indicate that the depletion of PLOD2 could inhibited EMT in cervical cancer cells.

### PLOD2 is critical in TGF-β1-induced EMT and might promote the nuclear entry of β-catenin

**Fig. 5:**
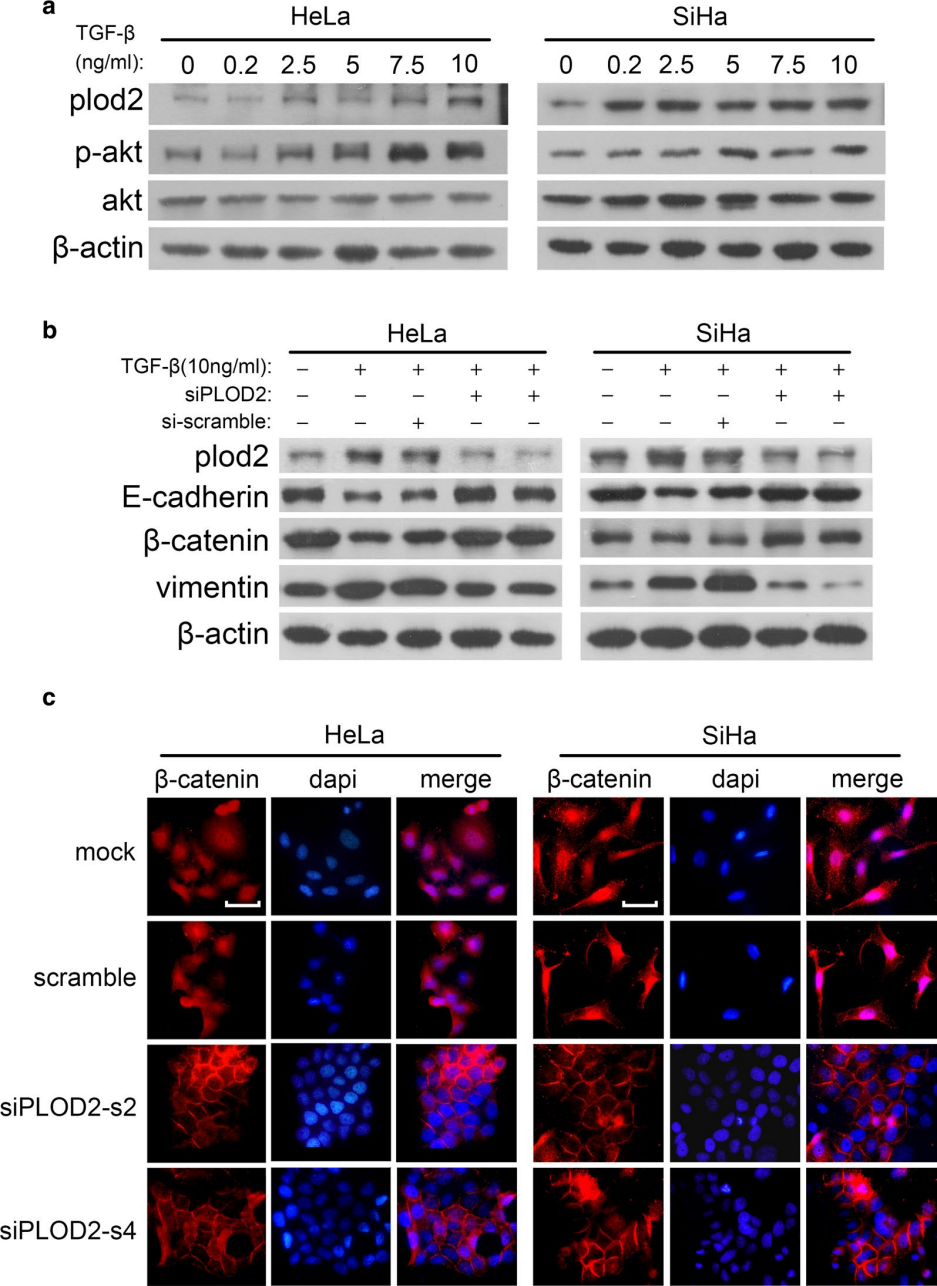
PLOD2 participates in TGF-β1-induced EMT by promoting the nuclear entry of β-catenin. **a** A Western blot showing an increase in PLOD2 expression after treatment with human TGF-β1 (concentration ranging from 0.2 to 10 ng/ml). The increase in p-AKT relative to total AKT was used to confirm the activation of TGF-β signalling. **b** Knockdown of PLOD2 attenuated TGF-β1-induced changes in EMT phenotype markers. Control cells and cells transfected with siPLOD2 treated with human TGF-β1 (10 ng/ml) for 72 h were subjected to Western blotting to detect EMT phenotype markers; β-actin was used as a loading control. **c** The depletion of PLOD2 inhibits the nuclear translocation of β-catenin induced by TGF-β1. After 72 h of treatment with TGF-β1, control cells exhibited strong β-catenin nuclear accumulation (*red*), whereas β-catenin accumulated in the membranes of siPLOD2 cells. DAPI was used to indicate nuclei (*blue*). Photos were captured under an oil lens (×1000)

EMT is a multi-step process that is accompanied by abnormal gene expression and aberrant cell signalling. Previous studies have shown that EMT can be triggered by the interplay of ECM components and soluble growth factors [40]. Among these factors, TGF-β1 has emerged as a potent driver of cancer progression, and this effect is mediated by the induction of EMT [42]. Specifically, TGF-β1 recruits histone-modifying enzymes to the PLOD2 promoter in a sp1- and smad3-dependent manner [43]. Treating HeLa and SiHa cells with 0.2–10 ng/ml human TGF-β1 validated the induction of PLOD2 by TGF-β1, as demonstrated by Western blotting (Fig. 5a). This TGF-β1 pathway activation was confirmed by the increase in phosphorylated-AKT relative to total-AKT [44], as also shown by Western blotting (Fig. 5a).

As mentioned above, knockdown of PLOD2 resulted in morphological changes (Fig. 4a, b) and changes in molecular markers (Fig. 4c, d) in HeLa and SiHa cells, which suggested that PLOD2 participates in the regulation of EMT. Taken together, these findings indicate that TGF-β1-induced EMT is partly mediated by PLOD2. To confirm this hypothesis, we treated cells with human TGF-β1 (10 ng/ml) for 72 h after knockdown of PLOD2 and then quantitated changes in molecular markers indicative of EMT. Western blotting showed that TGF-β1-treated but PLOD2-depleted cells exhibited decreases in epithelial markers, such as E-cadherin and β-catenin, and an increase in the mesenchymal marker vimentin compared with cells treated only with TGF-β1 (Fig. 5b). These results showed that PLOD2 inhibited TGF-β1-induced EMT.

A growing amount of evidence suggests that an increase in the transcriptional activity of β-catenin correlates with EMT [45, 46], and a loss of E-cadherin often up-regulates β-catenin signalling [47]. Because PLOD2 overexpression down-regulated E-cadherin in this study, we inferred that PLOD2 may be involved in the activation of β-catenin signalling. To test this hypothesis, we examined the nuclear localization of β-catenin. Immunofluorescence studies showed that stimulation by TGF-β1 for 72 h resulted in the robust nuclear accumulation of β-catenin in control cells, whereas β-catenin accumulated at the cell membrane in cells pre-transfected with siPLOD2 (Fig. 5c). These data suggest that PLOD2 is required for TGF-β1-induced EMT, and this effect is in part mediated by the inhibition of β-catenin nuclear translocation.

### PLOD2 affects the adhesion of cells to the ECM via an FAK-dependent mechanism

**Fig. 6:**
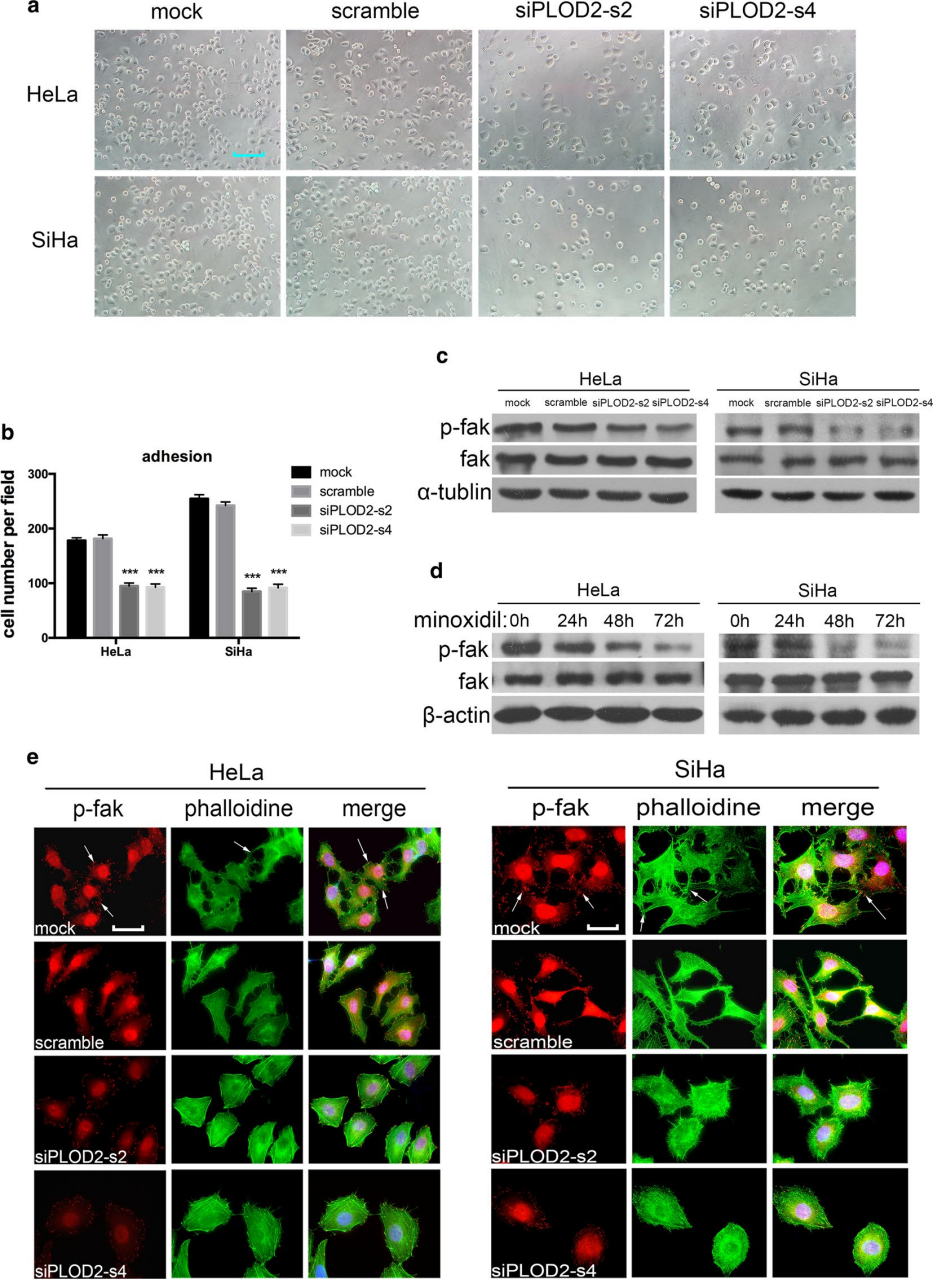
PLOD2 is required for the adhesion interactions necessary for migration and invasion and acts by promoting the formation of focal adhesions. **a** The adhesion of HeLa and SiHa cells to matrix assessed 2 h after plating. Representative phase-contrast images of control and siPLOD2 cells are shown. **b** Statistical analysis of adhesion (**a**) based on the mean ± SD of at least three independent experiments. *p* values were obtained using Student’s t test. *p* < 0.05 is indicated by “**”, and *p* < 0.001 is indicated by “***”. **c**, **d** Western blots showing the expression of total FAK and phospho-FAK in control cells and siPLOD2 cells (**c**) or cells treated with minoxidil (0.5 mM) (**d**). **e** P-FAK immunofluorescent staining and phalloidin staining of HeLa and Siha cells. *Arrows* indicate focal adhesions, as confirmed by co-staining for p-FAK. Note the intense staining at the leading edge of invasive pseudopod protrusions, indicated by *arrows*. Photos were captured under an oil lens (×1000)

Increased adhesion is a characteristic of invasive cells with a mesenchymal phenotype and is essential for the motility of these cells. Both HeLa and SiHa cells transfected with PLOD2 siRNA showed diminished adhesion to type I collagen gel (Fig. 6a), and a statistical analysis validated that this effect was significant when comparing control cells and siPLOD2 cells (Fig. 6b). The acquisition of motility is required before cells can migrate and invade. Invasive cell migration is a multi-step process that commences with pseudopod protrusion at the leading edge driven by actin polymerization, resulting in focal adhesion formation and the activation of integrins and phosphorylation of FAK [48].

We observed intense immunofluorescent staining for p-FAK (Red) and hair-like fibres stained with phalloidin (Green) protruding from cell surfaces into the collagen matrix, especially assembled at the leading edge of control cells, whereas pseudopod protrusion was minimal in PLOD2 siRNA cells, accompanied by dull p-FAK staining (Fig. 6e). Western blotting showed decreases in phosphorylated FAK compared with control cells when PLOD2 was knocked down by siRNA (Fig. 6c) or inhibited by minoxidil (Fig. 6d), whereas total FAK expression remained unchanged. Furthermore, we noted actin cytoskeleton remodelling and an increase in stress fibre and focal adhesion formation in control cells compared with cells transfected PLOD2 siRNA (Fig. 6e). Taken together, these results demonstrate that PLOD2 plays a crucial role in cell motility by affecting cell adhesion via FAK activation and cytoskeleton reconstruction.

### PLOD2 expression is associated with human cervical cancer progression

**Fig. 7:**
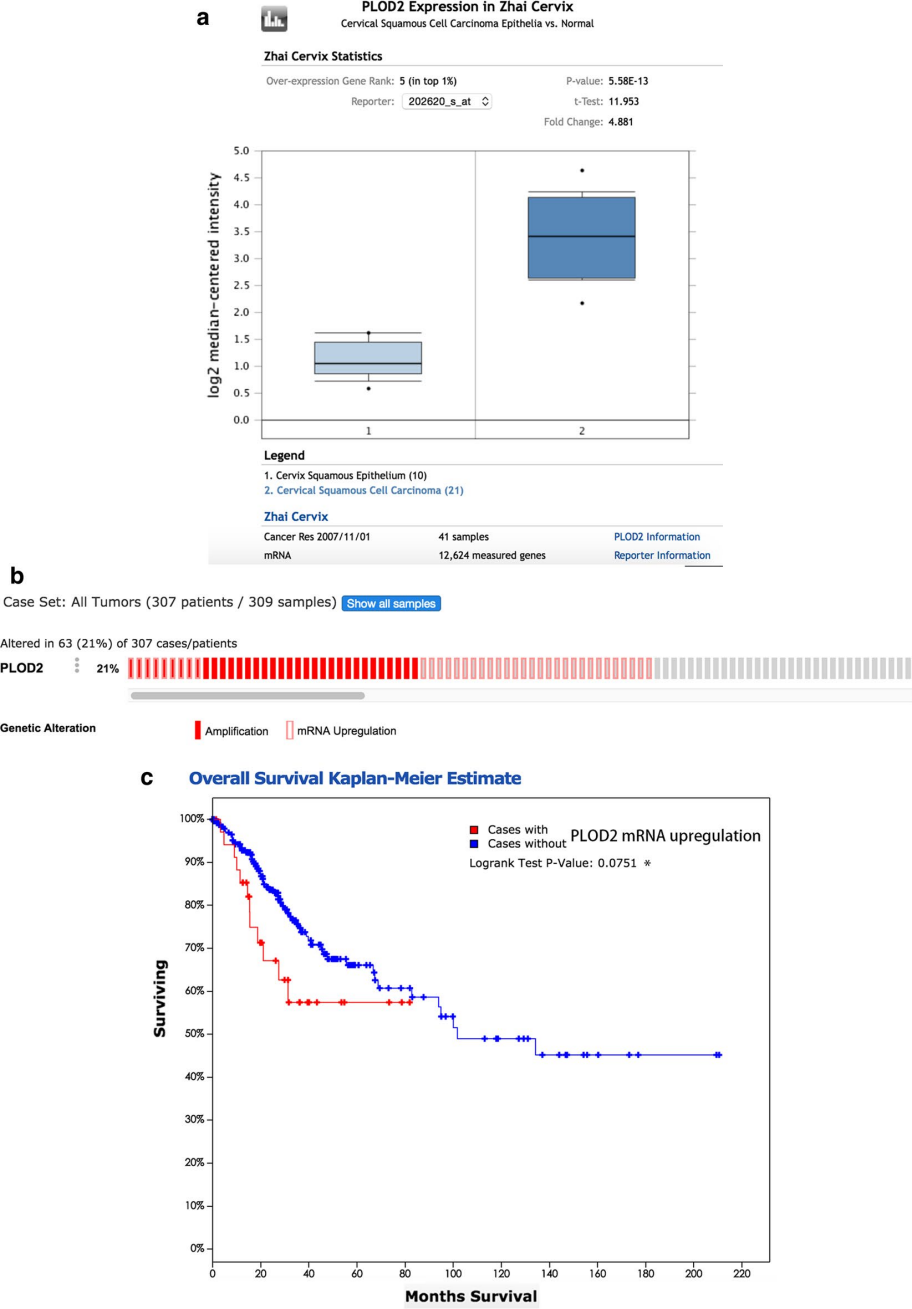
PLOD2 is up-regulated in cervical cancer. **a** Comparison of the expression of PLOD2 between cervix squamous epithelium and squamous cell carcinoma samples in the Zhai Cervix database using Oncomine. **b** Genetic and transcriptional alterations in PLOD2 in a TCGA cervical cancer dataset analysed using cBioPortal. **c** Kaplan–Meier analysis of the overall survival of 307 patients stratified by PLOD2 mRNA expression above the median level or below the median level using cBioPortal

To investigate the clinical significance of procollagen lysyl hydroxylase expression in cervical cancer, we compared PLOD2 gene expression in normal human cervix and cervical cancer tissues using the Oncomine database (http://www.oncomine.org). An analysis of a representative data set (Zhai cervix) revealed that PLOD2 mRNA expression levels were significantly higher in cervical squamous cell carcinoma than in cervix squamous epithelium (Fig. 7a). The results were corroborated when we interrogated The Cancer Genome Atlas (http://tcga-data.nci.hih.gov) for PLOD2 expression in cervical cancer. Specifically, PLOD2 mRNA was up-regulated in 20% of 307 patients with cervical cancer (Fig. 7b). Kaplan–Meier curves of overall survival stratified by PLOD2 mRNA levels in this dataset revealed that high PLOD2 expression was significantly associated with decreased overall survival (*p* = 0.0751) (Fig. 7c). These data indicate that PLOD2 expression is up-regulated and specifically prognostic in cervical cancer.

## Discussion

Emerging data suggest that hypoxia and the ECM play essential roles in metastasis, and they were originally considered independent contributors to metastatic spread. However, recent studies have established a direct link between tumour hypoxia and the composition and organization of the ECM [49]. Therefore, the molecules that link these mechanisms warrant further exploration to understand how hypoxia and ECM remodelling converge to synergistically promote cancer progression. PLOD2 has been firmly established to remodel the ECM, and hypoxia significantly induces PLOD2 expression, as mentioned above.

In the realm of cancer research, PLOD2 has been thoroughly explored in several studies and was confirmed to mediate hypoxia-induced cancer metastasis via collagen modification and ECM remodelling in primary tumours [14, 16, 26]. Specifically, the up-regulation of PLOD2 in tumour cells and tumour stromal fibroblasts changes collagen cross-linking tumour stroma to form aligned and stiff collagen fibres. These networks act as “highways” for tumour cells by supporting their scaffold and facilitating their migration towards blood vessels to result in ultimate dissemination to distant sites [50–53]. In addition to tumour cells, stromal cells, such as fibroblasts, also contribute to the up-regulation of PLOD2 in hypoxic breast tumour and finally lead to the remodelling of the ECM [29]. Moreover, PLOD2 participates in tumour cell-microenvironment communication. Specifically, PLOD2 depletion in cancer-associated fibroblasts (CAFs) reportedly abrogate the ability of them to promote tumour cell invasion and migration in vitro and in vivo [54].

In addition to the previously reported mechanism mediated by PLOD2 in other cancer types, we elucidated its influence on the typical malignant behaviour of cervical cancer cells, which affects several mechanisms that regulate cancer metastasis, such as cell adhesion, migration and invasion. Specifically, we showed that hypoxia increases PLOD2 expression to enhance the migration and invasion of HeLa and SiHa cells, and the inhibition of PLOD2 rescued the hypoxia-induced increase in cell motility. In addition, transforming growth factor-β1 up-regulated PLOD2, which might participate in TGF-β1-induced EMT. Finally, PLOD2 activated FAK to foster the formation of focal adhesions, which are essential for cell motility. Because radiotherapy exacerbates hypoxia in the tumour microenvironment, our findings might contribute to individual treatment strategies, especially for patients who have experienced relapse after surgery.

However, we did not thoroughly explore the function of PLOD2 in cervical cancer in our study. First of all, the PLOD2-mediated remodelling of the ECM has not been investigated in cervical cancer, which is a complex progress involving multiple cellular components and numerous cell biological process related to tumour progression. According to the research about PLOD2 in fibroblasts and CAFs, we propose that it probably participate in the communication of tumour cells with fibroblasts in the tumour stroma, which also deserves further exploring in cervical cancer. Second, we showed that PLOD2 is critical in TGF-β1-induced EMT progression and might promote the nuclear entry of β-catenin. Furthermore, as shown in Fig. 5a, the expression of p-AKT relative to total-AKT increased along with the induction of PLOD2 in cells treated with TGF-β. AKT can phosphorylate β-catenin directly or by inactivating GSK-3β [55]. The phosphorylation of β-catenin promotes its accumulation in the nucleus and its transcriptional activity [56]. Moreover, the activation of AKT results in EMT by down-regulating various epithelial-specific proteins, including β-catenin and E-cadherin [57]. Therefore, we assessed the effect of PLOD2 on the phosphorylation of AKT in HeLa and SiHa cells and determined that the knockdown of PLOD2 reduced the expression of p-AKT relative to total-AKT (Additional file 2: Fig. S2a). Furthermore, the inhibition of PLOD2 by minoxidil had similar results (Additional file 2: Fig. S2b). Therefore, we proposed that PLOD2 promotes the nuclear entry of β-catenin by activating AKT. However, the specific molecular mechanism underlying this effect remains unknown and warrants further exploration. Third, we herein investigated the effect of PLOD2 on two types of cervical cancer cells, and the overall effects of PLOD2 were more evident in the squamous cell carcinoma cell line (SiHa) than the adenocarcinoma cell line (HeLa). As we known, cervical adenocarcinoma confers a worse prognosis than squamous cell carcinoma, with higher rates of lymph node involvement and distant metastases [58, 59]. Furthermore, the two histological types of cancer have distinct molecular profiles [60]. Accordingly, the difference in the effect of PLOD2 on HeLa and SiHa cells in our work supports that the molecular mechanism by which PLOD2 affects pathogenesis of cervical cancer depends on the histological subtype and suggests that PLOD2 might be associated with genes that differentially expressed in these two histological types of cervical cancer. Several studies also demonstrated that the prognostic value of individual tumour markers differs by cervical cancer histological subtype [61]. Similarly, we speculate that PLOD2 may be involved in different gene regulation networks or participates in distinct biological process according to the histological subtype. Therefore, further studies are required to establish the role of PLOD2 in different histological types of cervical cancer. This will also help to identify patients who may benefit from the inhibition of PLOD2 in an effort to provide more tailed therapy.

Collectively, these data indicate that PLOD2 warrants further exploration in cervical cancer and might be a potential biomarker that can predict metastasis and serve as a valuable therapeutic target for the prevention of metastasis in cervical cancer.

## Conclusions

The results of this study do support that PLOD2 regulates the migratory, invasive and adhesive capacities of cervical cancer cells. Hypoxia and TGF-β1 up-regulated PLOD2 to promote EMT and the formation of focal adhesions. Thus, PLOD2 may have therapeutic value in the prevention of cervical cancer metastasis.

## Additional files

**Additional file 1: Figure S1.** Knockdown of PLOD2 does not affect the protein expression of MMP2 and MMP9 in normal and hypoxia condition. **a** Western blot showing non-significant changes in MMP2 and MMP9 in siPLOD2 HeLa and SiHa cells compared with control cells. **b** The expression of MMP-2 and MMP-9 increase in all groups of cobalt chloride (150 μM) treated cervical cancer cells. However, there are no significant changes of MMP-2 and MMP-9 in cobalt chloride (150 μM) treated but siPLOD2 cells relative to cells only treated by cobalt chloride.

**Additional file 2: Figure S2.** Inhibition of PLOD2 suppresses the phosphorylation of AKT. **a** Western Blotting for the change of phosphor-AKT and total-AKT after the knockdown of PLOD2 by siRNA. **b** Western Blotting for the change of phosphor-AKT and total-AKT after treating cells with minoxidil (0.5 mM).

## Abbreviations

PLOD2: procollagen-lysine, 2-oxoglutarate 5-dioxygenase 2
TCGA: The Cancer Genome Atlas
EMT: epithelial-to-mesenchymal transition
qRT-PCR: real-time reverse transcription quantitative PCR
HIF-1α: hypoxia inducible factor1, α subunit
TGF-β1: transforming growth factor β1
ECM: extracellular matrix
DMEM: Dulbecco’s Modified Eagle’s Medium
FBS: fetal bovine serum
CAFs: cancer associated fibroblasts
MMP: matrix metalloproteinase
PBS: phosphate-buffered saline
