# Supplementary figures and images for: Hypoxia and TGF-β1 induced PLOD2 expression improve the migration and invasion of cervical cancer cells by promoting epithelial-to-mesenchymal transition (EMT) and focal adhesion formation
Source: Cancer Cell Int. 2017 May 12;17:54. doi: 10.1186/s12935-017-0420-z (PMC5427545; doi:10.1186/s12935-017-0420-z)

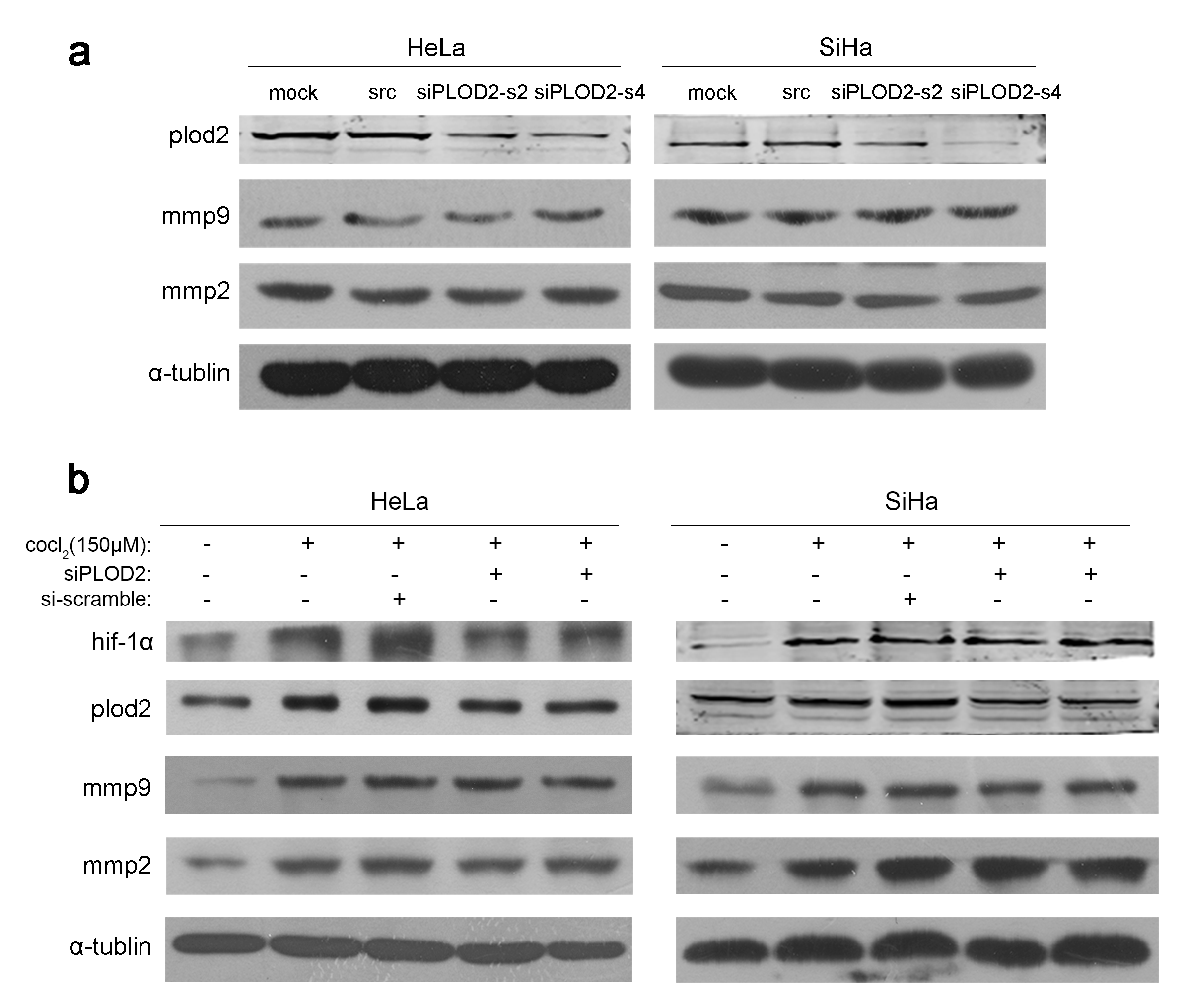

Supplement: Supplementary file 1 — Additional file 1: Figure S1. Knockdown of PLOD2 does not affect the protein expression of MMP2 and MMP9 in normal and hypoxia condition. a Western blot showing non-significant changes in MMP2 and MMP9 in siPLOD2 HeLa and SiHa cells compared with control cells. b The expression of MMP-2 and MMP-9 increase in all groups of cobalt chloride (150 μM) treated cervical cancer cells. However, there are no significant changes of MMP-2 and MMP-9 in cobalt chloride (150 μM) treated but siPLOD2 cells relative to cells only treated by cobalt chloride. [file 12935_2017_420_MOESM1_ESM.tif]

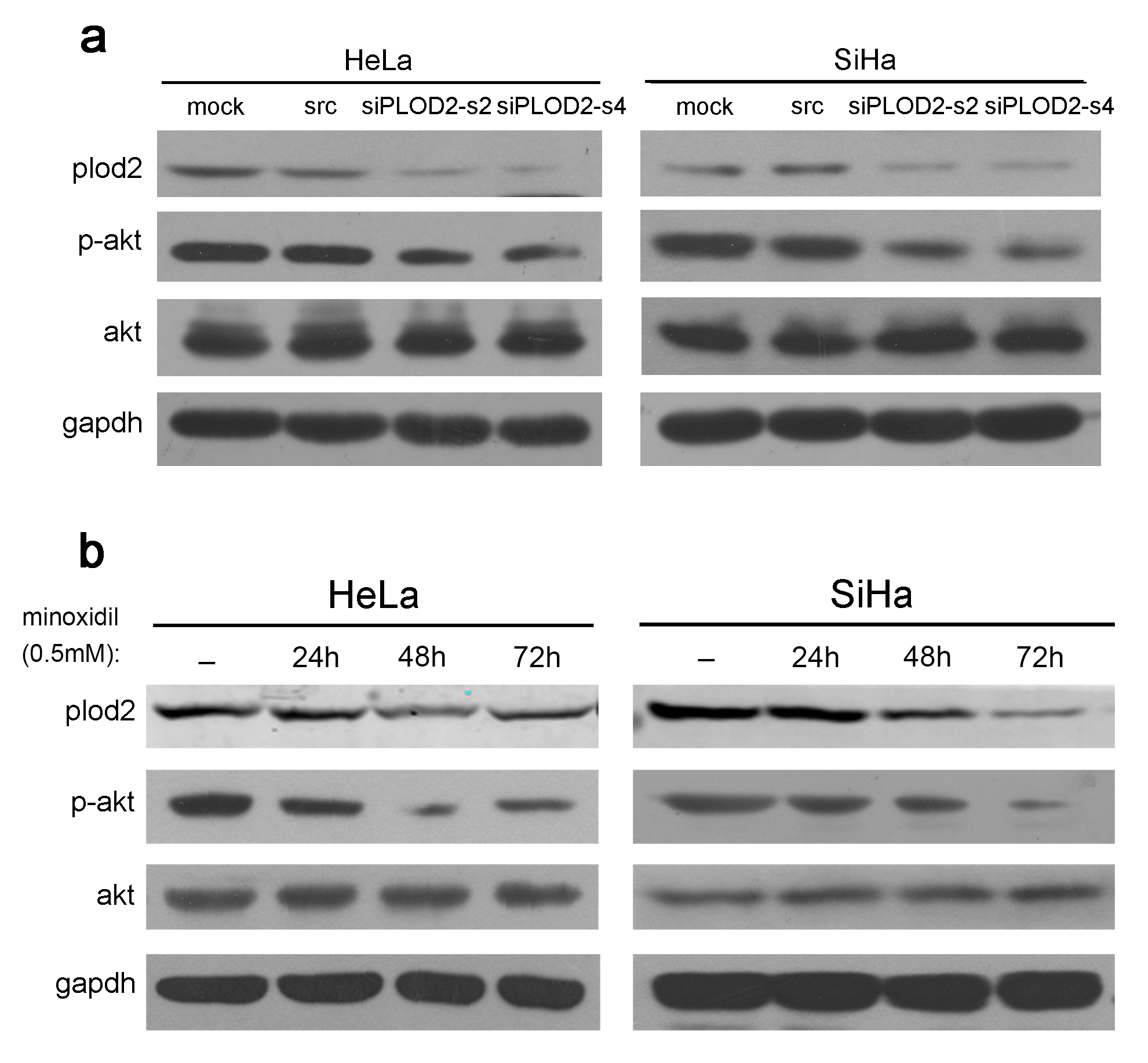

Supplement: Supplementary file 2 — Additional file 2: Figure S2. Inhibition of PLOD2 suppresses the phosphorylation of AKT. a Western Blotting for the change of phosphor-AKT and total-AKT after the knockdown of PLOD2 by siRNA. b Western Blotting for the change of phosphor-AKT and total-AKT after treating cells with minoxidil (0.5 mM). [file 12935_2017_420_MOESM2_ESM.tif]
